# Cystic Lesions of the Pancreas

**DOI:** 10.1155/1994/87365

**Published:** 1994

**Authors:** Johannes Scheele, Richard M. Charnley, Franz P. Gall

**Affiliations:** Department of Surgery University Hospital Erlangen Germany


### CYSTIC LESIONS OF THE PANCREAS

### ABSTRACT

*Warshaw, A. L, Compton, C. C., Lewandrowski, K., Cardenosa, G. and Mueller, P. R. (1990) Cystic tumors of the pancreas. New clinical, radiologic and pathologic observations in 67 patients. Annals of Surgery, 212, 432–445.*

**Within a 12-year period we treated 67 patients (49 women, 18 men; mean age, 61 years) with cystic neoplasms of the pancreas, including 18 serous cystic adenomas, 15 benign mucinous cystic neoplasms, 27 mucinous cystadenocarcinomas, 3 papillary cystic tumors, 2 cystic islet cell tumors, and 2 cases of mucinous ductal ectasia. Mean tumor size was 6 cm (2 to 16 cm). In 39% the patients had no symptoms, and in 37% the lesions had been misdiagnosed as a pseudocyst. Computed tomography was useful for detection, for distinguishing the microcystic subgroup of serous cystadenoma, and for showing rim calcification (all 7 cases were malignant) but was not reliable for distinguishing neoplasm from pseudocyst, serous from mucinous tumors, or benign from malignant. Arteriography showed hypervascularity in 4 of 10 serous adenomas, 3 of 11 mucinous carcinomas, and 1 of 1 papillar cystic tumors. Endoscopic pancreatography showed no communication with the cyst cavity in 37 of 37 cases of cystic neoplasms but opacified the ectatic ducts in 2 of 2 cases of mucinous ductal ectasia. Stenosis or obstruction of the pancreatic duct indicated cancer. The tumor was resected by distal pancreatectomy in 25 patients, by proximal resection in 29, and by total pancreatectomy in one, with no operative deaths. Forty-four percent of the tumors were malignant. In 10 cases the tumor was unresectable because of local extension or distant metastases, and those patients died at a mean of 4 months. Seventy-five percent of those resected for cure are alive without evident recurrence. Because the epithelial lining of the tumor was partially (5% to 98%) absent in 40% to 72% of cases of the major tumor types, and the mucinous component comprised only about 65% of mucinous cystadenoma lining, misdiagnoses on frozen and even permanent sections were made. Mitoses and histologic solid growth correlated with malignancy. Neuroendorine elements were seen in 87% of benign and 47% of malignant mucinous tumors. It is recommended that the terms macrocystic and microcystic be abandoned in favor of the histologic designations serous and mucinous. Incomplete examination of the cyst wall can be misleading, however. It is suggested that mucinous ductal ectasia be recognized separately from cystic tumors and that all of these lesions be resected, with the possible exception of asymptomatic confirmed serous cystadenomas.**

KEY WORDS: Pancreatic cystic tumours, Pancreas tumours

### PAPER DISCUSSION

Dr. Warshaw and his colleagues, certainly leading authorities on the subject of cystic neoplasms of the pancreas, have once again produced a paper of great interest. They have succeeded in giving us reliable guidelines on the diagnosis and treatment of this complex group of tumours in an article which demonstrates the depth of their experience and knowledge of pancreatic surgery.

Cystic tumours of the pancreas are not as common as pseudocysts but the distinction is vital[1,2]. Early adequate treatment of a malignant cystic neoplasm gives the patient a reasonable chance of cure whereas an inadvertent cystenterostomy reduces the prospects substantially.

A previous publication from Warshaw and Rutledge had focused on the distinction between neoplastic cysts (benign or malignant) and pseudocysts, based on both clinical parameters and imaging criteria, and is therefore essential reading for all clinicians involved in the management of patients with pancreatic disease[3]. Patients with neoplastic cysts usually have no history of acute or chronic pancreatitis nor of gallstones, alcohol abuse or trauma. Serum amylase is likely to be normal in neoplasia but raised in over half of the patients with a pancreatic pseudocyst. On imaging neoplastic cysts are more likely to be irregular or septate and may contain solid components. The wall is often calcified, calcification being confined to the cyst wall and not apparent in other parts of the pancreas which would be more likely in a pseudocyst due to the association with chronic pancreatitis[4]. Duct abnormalities characteristic of chronic pancreatitis may also be present in patients with pseudocysts where ERCP will identify cyst-duct communication in over 60% of patients. Such a communication would be exceptional in neoplastic cysts.

In this earlier publication Warshaw and Rutledge suggested that for diagnostic certainty aspiration of the cyst should be performed for amylase estimation and cytological examination. However, in the more recent article Dr. Warshaw has veered away from aspiration because of the risk of seeding of malignant cells in the needle track. We agree completely with this approach as we have found needle track seeding to be a real problem, particularly when biopsy is used for resectable malignant liver tumours[5]. To confuse the issue further aspiration of a neoplastic cyst may yield negative cytology and may even give an elevated amylase level[5,6]. We therefore prefer to rely upon those previously mentioned criteria to make a diagnosis and to provide a therapeutic strategy but would certainly not undertake percutaneous cyst aspiration if malignancy were a possible diagnosis.

We also share the reservations of the authors on the subject of biopsy of the cyst wall at the time of surgery. Absence of an epithelial lining must not be used to confirm the diagnosis of a pseudocyst since the epithelial lining is missing in many cases of cystic neoplasm. Frozen and even permanent section may fail to identify malignancy. If negative intraoperative histology is so unreliable, why should we then open a cyst to take a biopsy instead of resecting it in its entirety if we are doubtful as to its aetiology? A cyst-enterostomy should never be carried out in a patient where there may be a possibility that the lesion is neoplastic. The treatment of choice is resection! This may mean that rarely (one out of 68 patients in the experience of Warshaw and colleagues) patients with pseudocysts will undergo an inadvertent pancreatic resection. However, this is not such a disaster since resection is a reasonable treatment for large pseudocysts[2], and preferable to leaving behind a possibly curable tumour or creating needle track seeding. We do not feel that this is too radical an approach when Warshaw *et al.*, ourselves and others have shown that the mortality rate for pancreatectomy in the 1990s can be as low as 0–4%[7–10].

Within the group of cystic pancreatic neoplasms is it possible to distinguish benign from malignant lesions by preoperative imaging? Warshaw and colleagues have shown that CT and ultrasound are good techniques for detecting neoplastic cysts but rarely reliable for the prediction, or exclusion, of malignancy. For example, multiple small loculations are supposed to indicate a benign lesion but were found in only 50% of serous cystadenomas. A central scar with sunburst calcification is exclusively found in serous cystadenomas but was present in two cases only (11%). Nevertheless it may occasionally be used to 'confirm' the diagnosis of a benign lesion in a surgically high risk patient. In turn a peripheral rim of calcification on CT scan may indicate malignancy as all seven tumours in which it was seen turned out to be malignant. This should not be confused with pancreatic calcification seen in patients with pseudocysts secondary to chronic pancreatitis. Angiography was not found to be a useful discri-minator, neither was size useful. Both benign and malignant cysts varied in size from 2 to 16 cm and were unilocular or multilocular.

There are reports in the literature that cystadenocarcinomas have an extremely high cure rate[10]. Dr. Warshaw and colleagues, in this large series of patients, have a resectability rate of "only" 63% and corresponding overall cure rate of 48%. This compares with our own figure of curative resection for cystadenocarcinomas of approximately 60%. Although not a "high" cure rate, 48% is superior to results in selected subgroups of resectable periampullary cancer, and is very much better than the outcome of ductal adenocarcinoma of the pancreas. Nowadays when aggressive surgical resection of solid pancreatic cancer is becoming accepted as a reasonable therapeutic option it would be a great shame if cystic neoplasms, with their much better prognosis, were to be left undertreated with surgical conservativism and scepticism. As pointed out by Warshaw, we advocate that these patients should always undergo experienced surgical exploration with a view to pancreatic resection.

*References*

1. Layer, P. and Grandt, D. (1993) Diagnosis of pancreatic pseudocysts. In: Beger, H. J., Büchler, M. and Malfertheiner, P. (ed), Standards in pancreatic surgery. Springer-Verlag, Heidelberg-Berlin. 520–525
2. Grace, P. A. and Williamson, R. C. N. (1993) Modern management of pancreatic pseudocysts. *Br. J. Surg.*, **80**, 573–581
3. Warshaw, A. L. and Rutledge, P. L. (1987) Cystic tumours mistaken for pancreatic pseudocysts. *Ann. Surg.*, **205**, 393–398
4. Mullins, R. J., Mallangoni, M. A. and Bergamini, T. M. *et al.* (1988) Controversies in the management of pancreatic pseudocyst. *Am. J. Surg.*, **155**, 165–172
5. Scheele, J. and Altendorf-Hofmann, A. (1990) Needle track seeding from tumor biopsy on the liver. *Hepatogastroenterol*, **37**, 335–337
6. Railey, D. J., Barkin, J. J. and Livi, J. (1991) Aspiration of cystadeno carcinoma mimicking pancreatic pseudocyst. *Pancreas*, **6**, 491–492
7. Sachs, J. R., Deren, J. J., Sohn, M. and Nausbaun, M. (1989) Mucinous cystadenoma: pitfalls of differential diagnosis. *Am. J. Gastroenterol.*, **84**, 811–816
8. Geer, R. J. and Brannan, M. F. (1993) Prognostic indicators for survival after resection of pancreatic adenocarcinoma. *Am. J. Surg.*, **163**, 68–73
9. Gall, F. (1990) Das duktale Pankreaskarzinom. Langenbecks Arch Chir (Suppl II), **375**, 129–133
10. Cameron, J. L., Pitt, H. A. and Yeo, C. J., *et al.* (1993) One hundred and forty-five consecutive pancreaticoduodenectomies without mortality. *Ann. Surg.*, **217**, 430–438

Johannes Scheele, Richard M. Charnley
and Franz P. Gall
Department of Surgery
University Hospital Erlangen
F. R. G.

### HAS PROPRANOLOL RENDERED SCLEROTHERAPY OBSOLETE FOR POOR RISK ALCOHOLIC CIRRHOTIC PATIENTS?

### ABSTRACT

*Ink, O., Martin, T., Poynard, T., Reville, M., Anciaux, M.-L., Lenoir, C., Marill, J.-L., Labadie, H., Masliah, C., Perrin, D., Chaput, J.-C., Vetter, D., Eugene, C., Lebodic, L., Licht, H. and Etienne, J.-P. (1992) Does elective sclerotherapy improve the efficacy of long-term propranolol for prevention of recurrent bleeding in patients with severe cirrhosis? A prospective multicenter, randomized trial. Hepatology; 16: 912–919*

**We conducted a prospective, multicenter, randomized trial to compare the efficacy of sclerotherapy plus propranolol with that of propranolol alone in the prevention of recurrent gastroesophageal bleeding in severely cirrhotic patients. For 2 yr (1987 to 1988) 131 patients (96% of whom were alcoholic) with Child-Pugh class B or C cirrhosis (56% were class B and 44% were class C) were randomly assigned to one of our two treatment groups after cessation of variceal bleeding, without hemostatic sclerosis, and were observed for at least 2 yr. Treatment observance was good in 89% of cases; alcohol withdrawal was observed in 62% of cases. Sclerotherapy was performed weekly with 1% polidocanol, and variceal obliteration was obtained in 83% of cases, in a mean number of four sessions. The cumulative percentages (expressed as mean $\pm$ S.D.) of recurrent bleeding at 2 yr were 42% $\pm$ 6% for propranolol plus sclerotherapy and 59% $\pm$ 6% for propranolol alone (a nonsignificant difference). Twenty-eight patients from the propranolol group but only 12 patients from the propranolol-plus-sclerotherapy group had recurrent bleeding from esophageal variceal rupture ($p < 0.01$). The total number of blood units per patient with recurrent bleeding was slightly but not significantly more important in the propranolol group (8 $\pm$ 7) than in the propranolol-plus-sclerotherapy group (5 $\pm$ 5; $p = 0.09$). There were no statistical differences in the cumulative survival rate at 2 yr (propranolol plus sclerotherapy, 74% $\pm$ 6% and propranolol alone, 64% $\pm$ 6%) or in the number of patients who died of repeat bleeding (propranolol plus sclerotherapy, 13% $\pm$ 4% and propranolol alone, 17% $\pm$ 5%). Among the surviving patients, cirrhosis**

